# Management of Giant Ventral Hernia by Polypropylene Mesh and Host Tissue Barrier: Trial of Simplification

**DOI:** 10.4021/jocmr2009.10.1268

**Published:** 2009-10-16

**Authors:** Samir A. Ammar

**Affiliations:** a Surgery Department, Assiut University Hospitals, El Gamma Street, Assiut, Egypt. Email: microsurg1@gmail.com

## Abstract

**Background:**

Surgical management of giant ventral hernias is a surgical challenge due to limited abdominal cavity. This study evaluates management of giant ventral hernias using polypropylene mesh and host tissue barrier after suitable preoperative preparation.

**Methods:**

In the period from January 2005 and January 2007, 35 patients with giant ventral hernias underwent hernia repair. After careful preoperative preparation, repair was done using polypropylene mesh. The mesh was separated from the viscera by a small part of the hernia sac and the greater omentum.

**Results:**

The average age of the patients was 52. Twenty patients had post-operative incisional and 15 had para-umbilical hernias. The mean hernia defect size was 16.8 cm. Mean body mass index was 33. Follow up ranged from 18-36 months. No patient required ventilation after operation. Recurrent seroma, which responded to repeated aspiration, was experienced in 4 patients. Minor wound infection was observed in 5 patients. Small hernia recurrence occurred in one patient.

**Conclusion:**

The use of polypropylene and host tissue barrier after suitable preoperative preparation is relatively simple, safe, and reliable surgical solution to the problem of giant ventral hernia.

**Keywords:**

Hernia repair; Giant ventral hernia; Polypropylene mesh

## Introduction

The management of giant hernia with loss of abdominal domain remains a surgical challenge. Loss of abdominal domain occurs when the intra-abdominal contents can no longer lie within the abdominal cavity [1]. Giant ventral hernias are considered in cases where the hernia orifice is greater than 10 cm [2]. Huge hernias are more liable to complications and poorly controlled by external support. There are many problems associated with the management of such giant hernias. Firstly reduction of the contents is difficult. Postoperative disorders in the cardiovascular system, tissue oxygenation, increased intraabdominal pressure, and pulmonary embolism expose the patient to severe risks [3,4]. As the hernia is large, the risk of recurrence is high. Lastly the residual skin needs excision for cosmetic reasons.

The objective of this study is to evaluate management of giant ventral hernia using polypropylene mesh and host tissue barrier after appropriate preoperative preparation. For tension free closure, no attempt at approximation of the muscle to close the defect was done. The mesh was separated from the viscera by a host tissue barrier composed of a small part of the hernia sac and the greater omentum.

## Patients and Methods

This study is a prospective study included 35 patients treated at surgery department Assiut University Hospital in the period from January 2005 and January 2007.

Patients booked for elective repair of giant ventral hernia had complete preoperative fitness. All patients gave written informed consent, and the local ethics committee approved the study. The exclusion criteria were as follows: strangulated or obstructed hernia, major co-morbidities as severe cardiac, renal or respiratory disease, and intra-operative musculoaponeurotic defect 10 cm or less in diameter. Two days before surgery, the patients kept on a low residue diet that changed to full liquid diet the day before operation. The patients underwent colonic washing out using Sodium Phosphate enema to deflate the bowel the day before surgery.

All procedures were done under general endotracheal anesthesia. At the onset of anesthesia, a cephalosporin was administered intravenously. A nasogastric tube and Foleys catheter were introduced after induction of anesthesia. An elliptical skin incision was done incorporating any redundant skin and fat. The incision was deepened laterally to expose the musculoaponeurotic abdominal wall of at least 6 cm from the margin of the defect. The sac of the hernia was often quite large, long, and multilocular. The sac was opened and its surface is cleared off all adherent omentum and intestine. Most of the sac was excised except a small part that is used as a flap to close the defect over the replaced content. Where possible, the omentum was spread over the reduced bowel. No attempt at approximation of the muscle to close the defect was done. After securing hemostasis, a polypropylene mesh was inserted to cover the area so that at least 3 to 5 cm of the mesh overlapped the edges of the fascia and sutured to the outer surface of musculoaponeurotic abdominal wall (onlay). All redundant skin and fat were removed before insertion of two suction drains and skin closure.

The patients were closely observed postoperatively for adequate pain control, urine output, and blood gases. As soon as practical, the patient was raised to about 45-degree flexion of the trunk in order to allow maximum pulmonary ventilation. The intravenous infusion was continued until return of bowel sounds. Semisolid and solid diets were then gradually advanced. The patient remained catheterised until he/she can get out of bed. The drains were removed when the output was less than 30 cc within 24-hours period.

### Statistics

Data were described by using descriptive statistics as range, mean, standard deviation (SD) and percentage.

## Results

**Figure 1:**
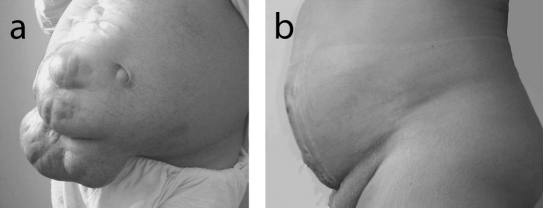
(a) Preoperative view of giant postoperative incisional hernia; (b) Appearance of the patient after hernial repair by polypropylene mesh and host tissue barrier.

**Figure 2:**
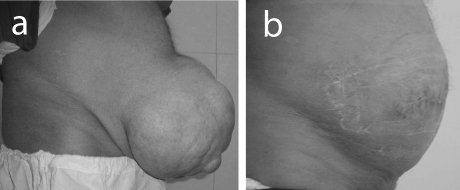
(a) Preoperative view of giant paraumbilical hernia; (b) postoperative view of the same patient.

The study included 35 patients with giant ventral hernias, 22 men and 13 women. The average age of patients was 52 years (SD = 5.27). Twenty had giant post-operative incisional hernias (Fig. 1) and 15 patients had giant para-umbilical hernias (Fig. 2). The mean hernia defect size was 16.8 cm (SD = 3.4). All patients were overweight or obese with a mean body mass index 33 (SD = 4.3). Follow up ranged from 18 to 36 months. All patients were discharged home within 7 - 15 days. No patient required ventilation after operation. Recurrent seroma, which responded to repeated aspiration, was experienced in 4 patients (11.4%). Minor wound infection was observed in 5 patients (14.2%). Small hernia recurrence occurred in one patient (2.8%). The recurrence was asymptomatic and the patient declined re-operation.

## Discussion

All hernias, particularly the massive one, should be repaired unless the patient is unable or unwilling to undergo surgery. Hernias increase gradually in size, unsightly, and are liable for grave complications. There are many options proposed to help repair of massive hernias.

For abdominal rooming, musculoskeletal flaps [5-7] and pneumoperitoneum [8,9] are described. Pneumoperitoneum is an invasive procedure with occasional complications, such as viscera perforation, air embolism, peritonitis, and hematoma of the abdominal wall [9-11]. Musculoskeletal flaps require much dissection with the possibility of significant blood loss, flap necrosis and donor site related complications [6].

To decrease the bulk of the contents, parts of omentum, small bowel or colon are resected [12]. However, bowel resection contaminates the field and is liable for serious complications. Historically, the use of synthetic mesh in the presence of potential contaminations has been strongly discouraged on the basis of high rates of morbidity [13,14].

Other options include using the components separation technique initially described by Ramirez et al in 1990 [15]. One of the limitations of this technique is that it requires the availability of viable local tissue to provide the necessary advancement for a durable repair. Often, massive ventral hernias do not contain sufficient surrounding tissue necessary to perform a component separation during the initial procedure and synthetic mesh is usually needed [16]. Alternatively expansion of available tissue over several weeks could be done using implantable tissue expanders to achieve primary closure [17]. After sequential expansion, the defect is typically closed by using a mesh repair. This method is further limited by the cost of the expanders and infection possibility. Staged repair of massive hernia by serial excision of gore-tex mesh is another choice [18]. Although this technique provides anatomic closure by medialization of the rectus muscle, it needs multiple operations over several weeks.

Prosthetic mesh is widely used in the repair of ventral hernias. The use of sheets of non-absorbable mesh has revolutionized the repair of abdominal wall defects and rendered obsolete most of other older types of operations [10,19]. Mesh repair of the ventral hernia have superiority over suture repair with regards to the recurrence [20]. Polypropylene is most commonly used because it is easy to handle and relatively low in cost. Because polypropylene causes a pronounced inflammatory reaction, the mesh is well incorporated in the surrounding tissue of the abdominal wall. However, for the same reason, polypropylene causes a strong stimulus for the formation of adhesions [21-23].

Many physical barriers are used in closure of large abdominal defects to prevent contact of the non-absorbable mesh with bowel including the use of absorbable mesh as a screen or double mesh. However, it now appears that the absorbable mesh does not have any special characteristic as far as fewer adhesions and fistulae are concerned [10,24-26]. Other materials that act as a protective layer on the visceral side of the non-absorbable mesh have been introduced in surgery. The aim is to provide sufficient separation between the mesh and viscera while regeneration takes place. The use of anti-adhesive liquids as Sepracoat and Icodextrin solutions are investigated. Coating the polypropylene mesh with seprafilm or collagen or the use of physical barriers as human amniotic membrane are also studied [21,27-30]. However, the use of coatings or foreign physical barriers may increase the rate of mesh infection [28,30].

The possible complications when mesh comes into contact with the bowel include adhesions, chronic pain, bowel obstruction, and erosion into the bowel with enterocutaneous fistula formation [10]. None of these complications were experienced in this study. The contact between the bowel and the prosthesis was prevented by interposition a natural barrier. This barrier composed of the hernia sac and the greater omentum, both shield the bowel from contact with non-absorbable mesh. Careful Preoperative preparation by low residue diet, liquid diet, and colonic lavage is an important aid because it deflates the bowel. Aqueous sodium phosphate is a hyperosmotic solution draws plasma water into the bowel lumen to promote colonic cleansing. The ideal preparation would reliably empty the colon of fecal material, not cause any patient discomfort or harm and would be inexpensive [31].

To provide a larger abdominal cavity, no attempt to close the musculoaponeurotic defect was done. Therefore, the hernia contents can be replaced without tension and without compromising respiratory or cardiac functions. No patient required ventilation or suffered from compartment syndrome after operation in this series. The data in this study demonstrate low complications rate with the use polypropylene mesh and host tissue barrier in the repair of giant hernias. Small asymptomatic hernia recurrence occurred in one patient (2.8%). No significant infection, mesh exposure or fistulae were experienced. There was no need to remove any of meshes.

In conclusion, careful preoperative preparation, operative technique, and postoperative care are required for successful management of giant ventral hernias. The use of polypropylene and host tissue barrier is relatively simple, safe, and reliable surgical solution to the problem of giant ventral hernia and avoids extensive, staged, or costly operations.
